# Unilateral Thalamic Venous Infarction in an Infant: A Rare Presentation of Bilateral Deep Cerebral Venous Thrombosis

**DOI:** 10.1155/2018/3618619

**Published:** 2018-10-24

**Authors:** Katherine Chung, Umar Tariq, Rabia M. Khan, Thomas P. Nickles, Joseph H. Lock

**Affiliations:** ^1^Geisinger Commonwealth School of Medicine, Scranton PA 18510, USA; ^2^Department of Radiology, Geisinger Medical Center, Danville PA 17821, USA

## Abstract

Cerebral venous thrombosis (CVT) may manifest as superficial cerebral venous thrombosis (SCVT) or deep cerebral venous thrombosis (DCVT). Of the two patterns, DCVT is less commonly observed, although it often results in greater morbidity and mortality due to involvement of the deep gray nuclei. It can present at any age and typically results in edema of the bilateral thalami, with occasional extension into the basal ganglia. Unilateral thalamic infarct is rare and results in an ambiguous imaging pattern. We present the clinical and neuroimaging profile of an acute unilateral thalamic venous infarct in an infant secondary to bilateral DCVT. Early recognition of this atypical pattern will facilitate accurate diagnosis and treatment, and obviate the need for unnecessary interventions.

## 1. Introduction

Cerebral venous thrombosis (CVT) may be subdivided into superficial cerebral venous thrombosis (SCVT) or deep cerebral venous thrombosis (DCVT). Thrombosis of the deep system occurs in only 10% of CVT [1]. The deep venous system includes the basal and internal cerebral veins, great cerebral vein (of Galen) and straight sinus, which drain the deep gray nuclei. Because of this anatomy, DVCT typically results in bilateral thalamic infarction, with occasional extension into the basal ganglia. DCVT has a greater morbidity and mortality compared to SCVT due to involvement of the deep gray nuclei. We present a unique case of acute unilateral thalamic infarct secondary to bilateral DCVT in a pediatric patient with imaging and clinical correlation.

## 2. Case Presentation

A 15-month-old male presented to the Emergency Department (ED) with sudden onset of right arm and leg weakness beginning 3 hours prior to admission. His clinical history included a viral illness 5 days prior to admission, with malaise, fever, vomiting, and diarrhea. Early in the course of that illness he was seen by a pediatrician who noted mild dehydration, and suggested oral rehydration and antipyretics. He was otherwise healthy, with up-to-date immunizations.

**Figure 1:**
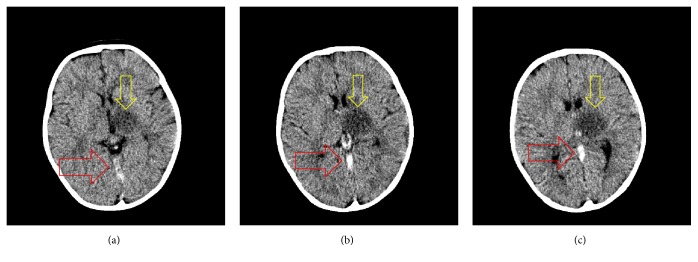
Axial noncontrast CT at the level of basal ganglia. These scans demonstrate dense clot sign which show a dark area (yellow arrows) where the infarct of the left thalamus is located due to thrombosis of internal cerebral veins and straight sinus (red arrows).

On arrival to the ED physical exam revealed flaccidity in right upper and lower extremities. X-rays of right upper extremity obtained to rule out trauma were negative. Lab results showed microcytic anemia with hemoglobin of 6 g/dL, and thrombocytosis, with a platelet count of 512,000. The remaining labs were normal. Computed tomography (CT) of the head without contrast showed hypodensity of the left thalamus (Figure 1). In addition, high attenuation was noted throughout the bilateral deep venous system, compatible with acute DCVT (Figure 1).

**Figure 2:**
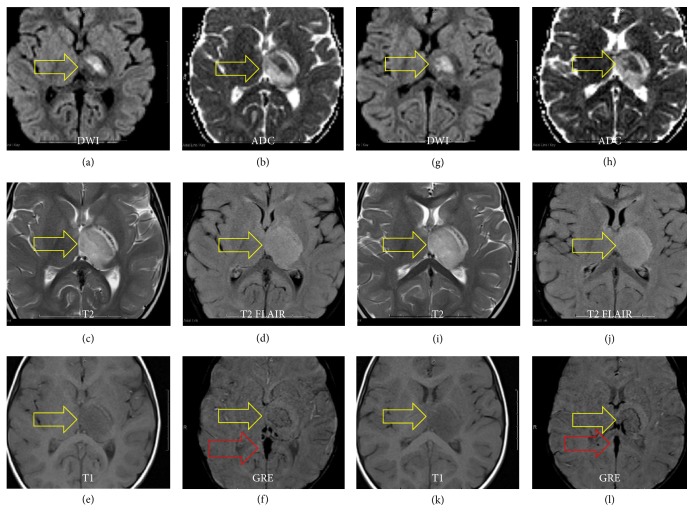
MRI obtained to confirm deep cerebral venous thrombosis and left thalamic infarction. DWI and ADC imaging ((a), (b), (g), and (h)) shows restricted diffusion in the anteromedial left thalamus without distinct territorial boundaries in relation to venous ischemia. T2 FLAIR hyperintensity ((c), (d), (i), and (j)) shows surrounding edema related to the infarction. GRE image (f) shows dark signal loss suggestive of petechial hemorrhage around the area of ischemia. GRE image (l) shows linear signal drop in the posteromedial surface of the left thalamus, highly suggestive of thrombus within a venous structure, likely part of the left internal cerebral vein.

**Figure 3:**
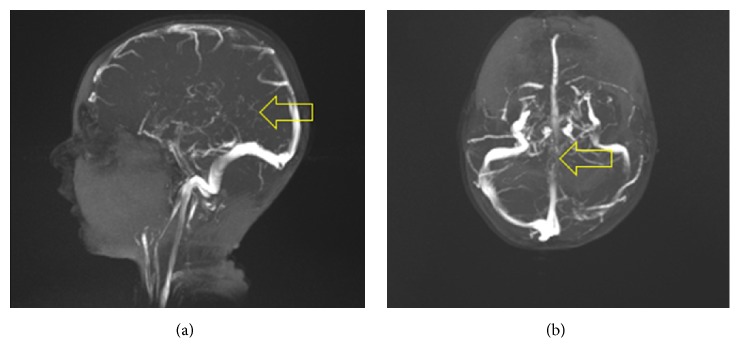
MRV was performed which showed a filling defect of straight sinus and internal cerebral veins (yellow arrow).

Anticoagulation therapy and IV hydration were initiated immediately after radiologic findings were discussed with the ED physician. The patient was transferred to the Intensive Care Unit of our tertiary pediatric hospital. Magnetic resonance imaging (MRI) of the brain demonstrated restricted diffusion in the central aspect of the thalamus, surrounded by vasogenic edema, compatible with acute venous infarction (Figure 2). No other parenchymal lesion was detected. Signal changes within the deep venous system were compatible with acute intraluminal thrombus (Figure 2). MR venography confirmed lack of flow-related signal throughout the deep venous system (Figure 3).

The patient had a follow-up MR venography done two days later before discharge but was found to have no significant interval change with relatively stable venous infarct in the left thalamus and posterior limb of the internal capsule. No other follow-up imaging was done since. His symptoms resolved completely after six months of physical and speech therapy without residual symptoms. He is now being followed closely by pediatric neurology and hematology physicians.

## 3. Discussion

DCVT is a rare cause of stroke which may affect the pediatric or adult population. Multiple risk factors have been associated with CVT. Dehydration, infection, thrombocytosis, and trauma have been described in the pediatric population. Genetic predisposition, malignancy, inflammatory bowel disease, pregnancy, and medications are additional factors seen in adults [2]. Anemia is a recently validated risk factor for CVT [3].

In cases of CVT or DCVT, treatment is initiation of anticoagulant after imaging confirmation of intraluminal venous thrombosis. The figures demonstrate edema and ischemia in relation to acute venous thrombosis. In contrast to arterial ischemic lesions, those tend to regress, with good recoveries if successful recanalization is promptly obtained. Our patient received IV heparin infusion and later transitioned to enoxaparin (Lovenox). He also received 15 mL/kg of packed RBCs and ferrous sulfate due to anemia. Children with CVT are at risk for developing seizures therefore prophylactic antiepileptics may be prescribed for up to one year. Patient remained on lovenox for 6 months.

Multiple labs, including a hypercoagulability panel, and clinical testing were done to establish the etiology of the patient's infarction. However, all were unremarkable except for a low ferritin (2.4 ng/nl) and a hemoglobin of 6 g/dL; anemia was suggested as a possible cause for thrombosis. Our patient also had a preceding viral URI complicated by dehydration. These factors were described as the inciting events for CVT in the consultation note from pediatric neurology.

Unilateral thalamic infarction is rare, but has been reported in the setting of unilateral internal cerebral vein thrombosis [4]. Four such cases have been reported, with left sided thromboses more frequent than right [4, 5]. To the best of our knowledge, our case is the first report of a unilateral thalamic infarct in the setting of bilateral DCVT.

The cerebrovascular system is complex because of vast variation in collateral circulation and brain structures and this case report serves as an example of this intricate, sophisticated system. We described a novel case of unilateral thalamic infarction secondary to bilateral DCVT. Interestingly, of the few case reports, unilateral thalamic infarct has been reported more frequently on the left side, although an underlying anatomic basis has not been proposed. We believe that there could be a possible collateral venous circulation to the right thalamus that causes the left thalamic infarction the more common presentation in setting of DCVT.
